# Surface hydrophobics mediate functional dimerization of CYP121A1 of *Mycobacterium tuberculosis*

**DOI:** 10.1038/s41598-020-79545-y

**Published:** 2021-01-11

**Authors:** Amit Kumar, Christopher S. Campomizzi, Natalie Jay, Shaun Ferguson, Emelie-Jo Scheffler, James Lioi, Chengjian Tu, Jun Qu, Claire Simons, D. Fernando Estrada

**Affiliations:** 1 Department of Biochemistry, Jacobs School of Medicine and Biomedical Science, University at Buffalo, Buffalo, NY 14203 USA; 2 Department of Pharmaceutical Sciences, School of Pharmacy, University at Buffalo, Buffalo, NY 14214 USA; 3 School of Pharmacy and Pharmaceutical Sciences, Cardiff University, King Edward VII Avenue, Cardiff, CF10 3NB UK

**Keywords:** Enzymes, Structural biology

## Abstract

Tuberculosis is caused by the pathogenic bacterium *Mycobacterium tuberculosis* (Mtb) and remains the leading cause of death by infection world-wide. The Mtb genome encodes a disproportionate number of twenty cytochrome P450 enzymes, of which the essential enzyme cytochrome P450 121A1 (CYP121A1) remains a target of drug design efforts. CYP121A1 mediates a phenol coupling reaction of the tyrosine dipeptide cyclo-L-Tyr-L-Tyr (cYY). In this work, a structure and function investigation of dimerization was performed as an overlooked feature of CYP121A1 function. This investigation showed that CYP121A1 dimers form via intermolecular contacts on the distal surface and are mediated by a network of solvent-exposed hydrophobic residues. Disruption of CYP121A1 dimers by site-directed mutagenesis leads to a partial loss of specificity for cYY, resulting in an approximate 75% decrease in catalysis. ^19^F labeling and nuclear magnetic resonance of the enzyme FG-loop was also combined with protein docking to develop a working model of a functional CYP121A1 dimer. The results obtained suggest that participation of a homodimer interface in substrate selectivity represents a novel paradigm of substrate binding in CYPs, while also providing important mechanistic insight regarding a relevant drug target in the development of novel anti-tuberculosis agents.

## Introduction

Tuberculosis (TB) is caused by the pathogenic bacterium *Mycobacterium tuberculosis* (Mtb) and remains the leading cause of death by infection world-wide. An estimated one-quarter of the world’s population is infected by Mtb, with approximately 10 million new cases reported annually by the World Health Organization (WHO, 2019). The combined effect of TB with Human Immunodeficiency Virus (HIV) is particularly lethal; approximately 250,000 of the world’s 1.5 million annual TB deaths are from patients previously infected by HIV^1^. Moreover, the rise of multidrug-resistant and extensively drug-resistant forms of the disease underscores the critical need to develop the next generation of anti-tuberculosis agents.

The Mtb genome contains a disproportionate compliment of 20 cytochrome P450 (CYP) genes^2^. By comparison, *E. coli*, with a genome of similar size, has no CYP genes, and human CYP gene density is approximate 1/200th that of Mtb. While the function of most Mtb CYPs remains an open question, gene knock-out targeting *rv2275* has been shown to result in nonviable bacteria, suggesting that the corresponding gene product, CYP121A1, is essential^3^. CYP121A1 mediates a phenol-coupling reaction on the tyrosine dipeptide cyclo-L-Tyr-L-Tyr (cYY) to form mycocyclosin^4^, a compound of as yet undetermined function.

Recent efforts at developing specific small-molecule inhibitors of CYP121A1 have centered around fragment screening and co-crystallization of fragment compounds and cYY mimics. This has led to a large number of CYP121A1 structures in a variety of liganded and unliganded states. Despite this wealth of structural data, there remain significant gaps in our understanding of CYP121A1 function. For example, the specific interactions between cYY and the large active site cavity are not entirely clear. The substrate co-crystallizes in a likely ligand access channel and without displacement of the heme-coordinated water molecule. This binding mode is not consistent with the Fe high-spin transition (type-I response) observed in cYY spectral binding assays^4^. Another open question, although one that has received far less attention, is whether CYP121A1 dimerization is functionally relevant. As has been reported previously, recombinantly produced CYP121A1 purifies as a dimer^5^. However, with only two exceptions (PDB 2IJ7 and 2IJ5) (https://www.rcsb.org/)^6^, the enzyme crystallizes primarily as a monomer. Therefore, the dimeric interface of CYP121A1 is currently unknown. To our knowledge, the only study to specifically address dimerization relied on nanospray ionization and mass spectrometry of intact native CYP121A1 to demonstrate that the introduction of the high-affinity azoles econazole and clotrimazole disrupts dimerization of the enzyme, thereby suggesting an association between dimerization and azole binding^5^.

In this study, how CYP121A1 dimer formation is related to its function was investigated. Glutaraldehyde cross-linking, coupled to mass spectrometry, was used to identify a hydrophobic dimer interface. Alanine substitutions of two surface-oriented isoleucine residues on the F and G α-helices resulted in the monomerization of the enzyme. Notably, the monomeric enzyme displayed a significant decrease in catalytic activity, likely stemming from compromised substrate specificity. Ligand-binding and thermal stability assays were also employed as well as comparative ^19^F nuclear magnetic resonance (NMR) of the FG-loop to characterize the functional dimer of the enzyme. These findings are discussed in the context of a modeled CYP121A1 homodimer complex. Moreover, these findings provide mechanistic insight that points toward dimerization as an underappreciated aspect of CYP121A1 function.

## Results

### Cross-linking and mass spectrometry

Purification of recombinant CYP121A1 produces the dimeric protein in solution^5,7^. A previous study reported that the presence of high-affinity azoles such as econazole redistributes the dimer equilibrium towards the monomeric enzyme^5^. However, our findings using a calibrated gel filtration column suggested that saturating CYP121A1 with econazole or the related inhibitor miconazole did not cause CYP121A1 to migrate on the column as a monomer (Supplementary Fig. S1). Therefore, to investigate the functional significance of CYP121A1 dimers, we first set out to determine the homodimer interface by chemical cross-linking coupled to chymotrypsin digest and mass spectrometry.

Purified CYP121A1 was first treated with glutaraldehyde. The capture of the dimer was verified by denaturing gel electrophoresis (Supplementary Fig. S2). Treated and untreated solutions of CYP121A1 were then subjected to proteolytic digest followed by separation of peptides by liquid chromatography with tandem mass spectrometry. In total, 18 peptides were quantified to a different abundance in comparison to samples that had not been cross-linked (Supplementary Table S1). These peptides overlap into four different primary amino acid sequences of CYP121A1 (Supplementary Fig. S2). Mapping of these four regions onto the CYP121A1 structure suggested a dimer interface that primarily involves the distal surface, which is acknowledged as the substrate-binding surface of CYP enzymes and is located on the side of the heme facing the active site cavity. Specifically, cross-linking occurred near the F and G α-helices, the B’-helix, and the β3 loop. In contrast, most of the proximal surface, which is located on the side of the heme that is opposite of the active site and is the site of CYP electron delivery occurs via redox partner binding, was not significantly affected, thus indicating that in the dimeric form, electron delivery is likely unimpeded.

### Engineering monomeric CYP121A1

**Figure 1:**
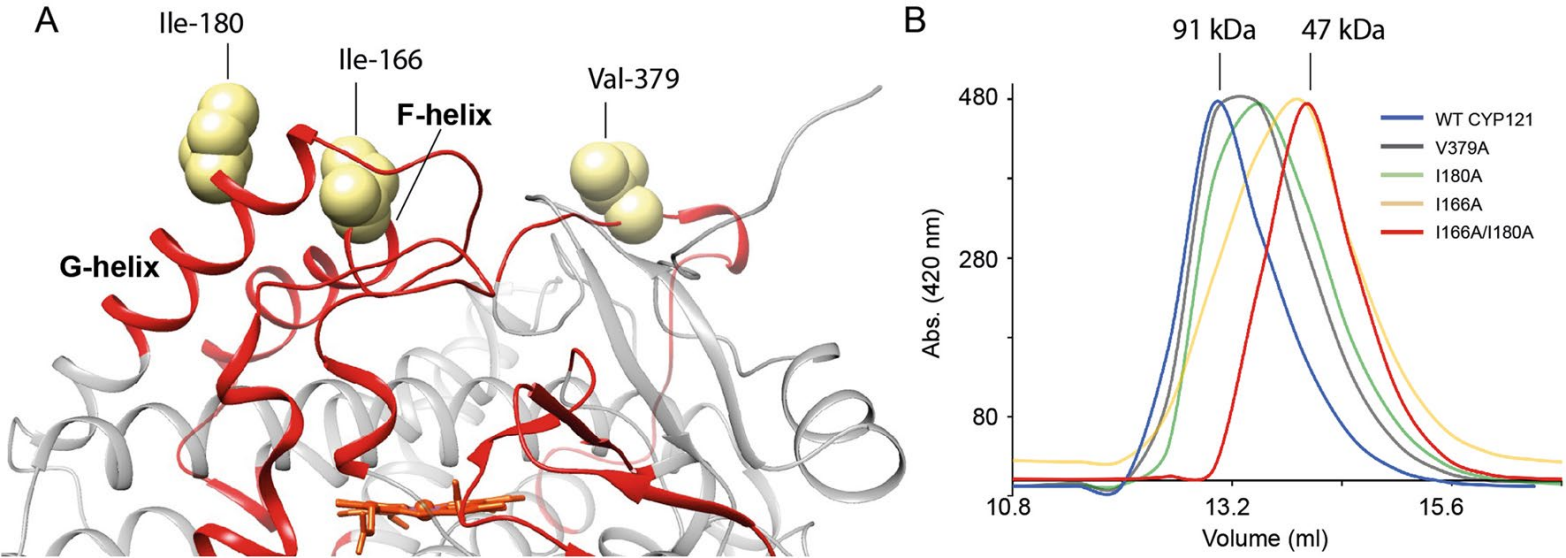
Engineering a monomer of CYP121A1. (**A**) Distribution of non-polar, solvent-exposed side chains on the distal surface of the protein. (**B**) Gel filtration column elution of WT and alanine mutations of CYP121A1. The figure in (**A**) was generated using UCSF Chimera v1.14 (www.cgl.ucsf.edu/chimera).

An analysis of surface amino acid residues in regions of CYP121A1 that are affected by glutaraldehyde cross-linking revealed three non-polar side chains that are solvent-exposed in all crystal structures (Fig. 1A). These include Ile-180 of the G-helix, Ile-166 of the F-helix, and Val-379 of the β3 loop. To examine the potential roles of these residues in homodimer formation, single and double mutant alanine substitutions were created. Three corresponding point alanine mutations were created, and owing to the proximity of Ile-180 to Ile-166, the double mutation I166A_I180A was also generated. All mutant forms of CYP121A1 expressed and purified similar to the wild-type protein. Purified samples were analyzed individually on a calibrated 24-ml gel filtration column under high salt conditions to prevent non-specific protein interactions. Follow-on glutaraldehyde cross-linking was also carried out using WT and the I166A_I180A form of the enzyme.

Despite similar cross-linking efficiency for monomeric and dimeric forms (Supplementary Fig. S3), by gel filtration, all proteins containing a single point mutation were found to migrate at a size consistent with a mixture of monomeric and dimeric CYP121A1 (Fig. 1B), with the order of elution from the column as; wild-type, V379A, I180A, and I166A. However, the I166A_I180A double mutant migrated on the column entirely as a monomer (calculated at 47 kDa) with no observable presence of dimeric protein. This finding confirms the role of the F and G helices in the dimer interface. Moreover, the observation that Val-379 also appears to disrupt the dimer equilibrium, albeit to a lesser extent than I166A or I180A, suggests that the interface is larger than the F and G α-helices alone and likely involves the β3 loop as well.

### CYP121A1 function is compromised in monomeric enzyme

**Figure 2:**
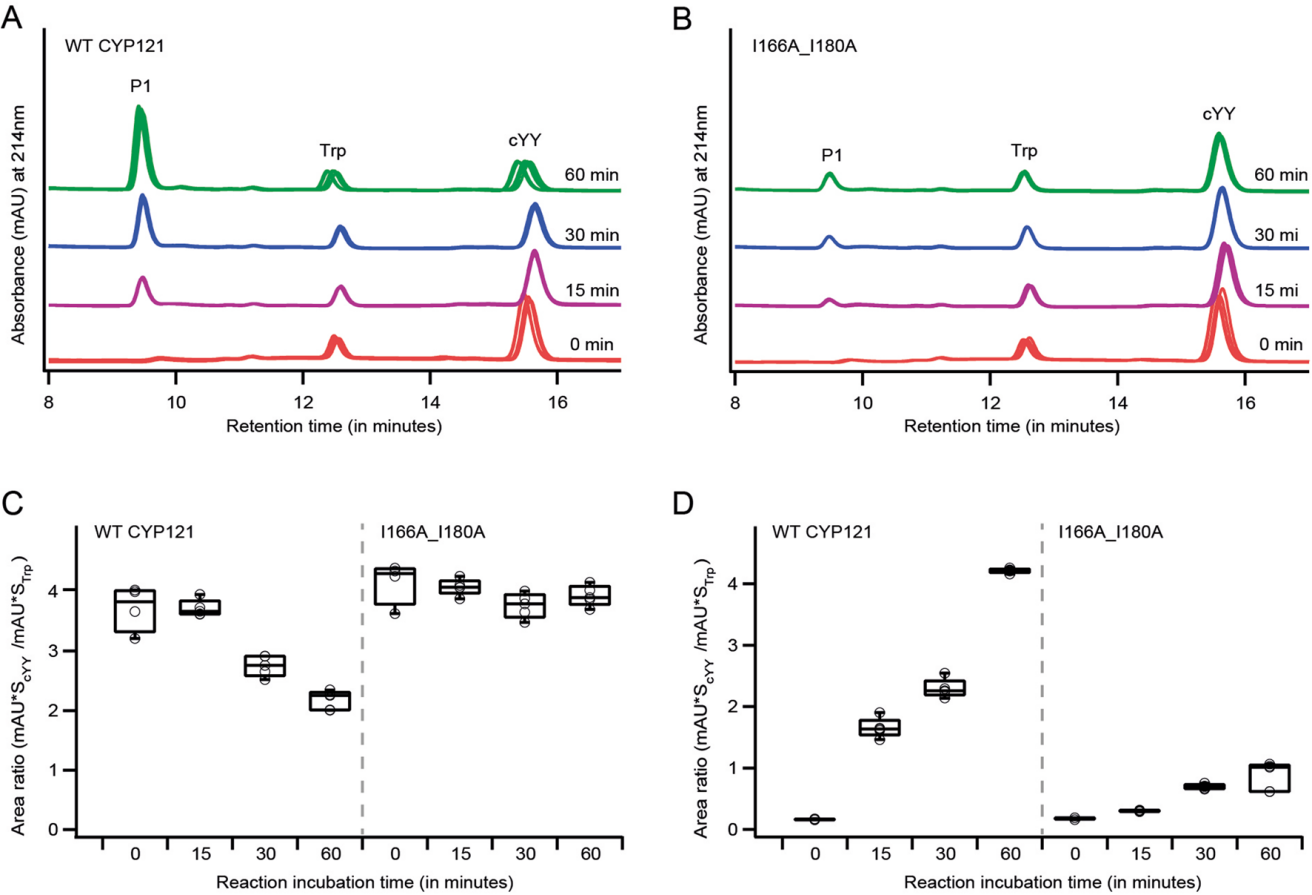
Time-course reconstituted CYP121A1 functional assays. A reconstituted functional assay using cYY as the substrate quenched at 0, 15, 30, and 60 min and resolved by using HPLC. (**A**) HPLC traces of replicates at different time points for (**A**) wild type CYP121A1 and (**B**) mutant I166A_I180A with increment in product formation (P1) and depletion in the substrate (cYY). Relative substrate depletion (**C**) and product formation (**D**) were measured using tryptophan as an internal standard. HPLC traces in (**A**) and Plots in (**B**) were generated using Igor Pro Version 6.37 (https://www.wavemetrics.com/) and graphics designed by Adobe Illustrator CS5 (https://www.adobe.com/).

In order to evaluate the functional impact of disrupting CYP121A1 dimers, comparative reconstituted functional assays for WT CYP121A1 and I166A_I180A were performed. For WT CYP121A1, reactions stopped after increasing incubation times and resolved by reverse-phase liquid chromatography showed a gradual increase in the elution of the product peak P at ~ 9.5 min (Fig. 2), paired with a decrease in the elution of cYY at ~ 15.5 min. The elution pattern was consistent with that reported previously for mycocyclosin and cYY^4^. Notably, after corresponding time periods, I166A_I180A showed approximately a ~ 75% decrease in product peak formation and minimal cYY depletion when compared to WT CYP121A1. Since no other product peaks were resolvable in the chromatographic trace, interpretation of these data would indicate a significantly compromised function of the I166A_I180A double mutant.

### CYP121A1 dimerization contributes to cYY specificity

**Figure 3:**
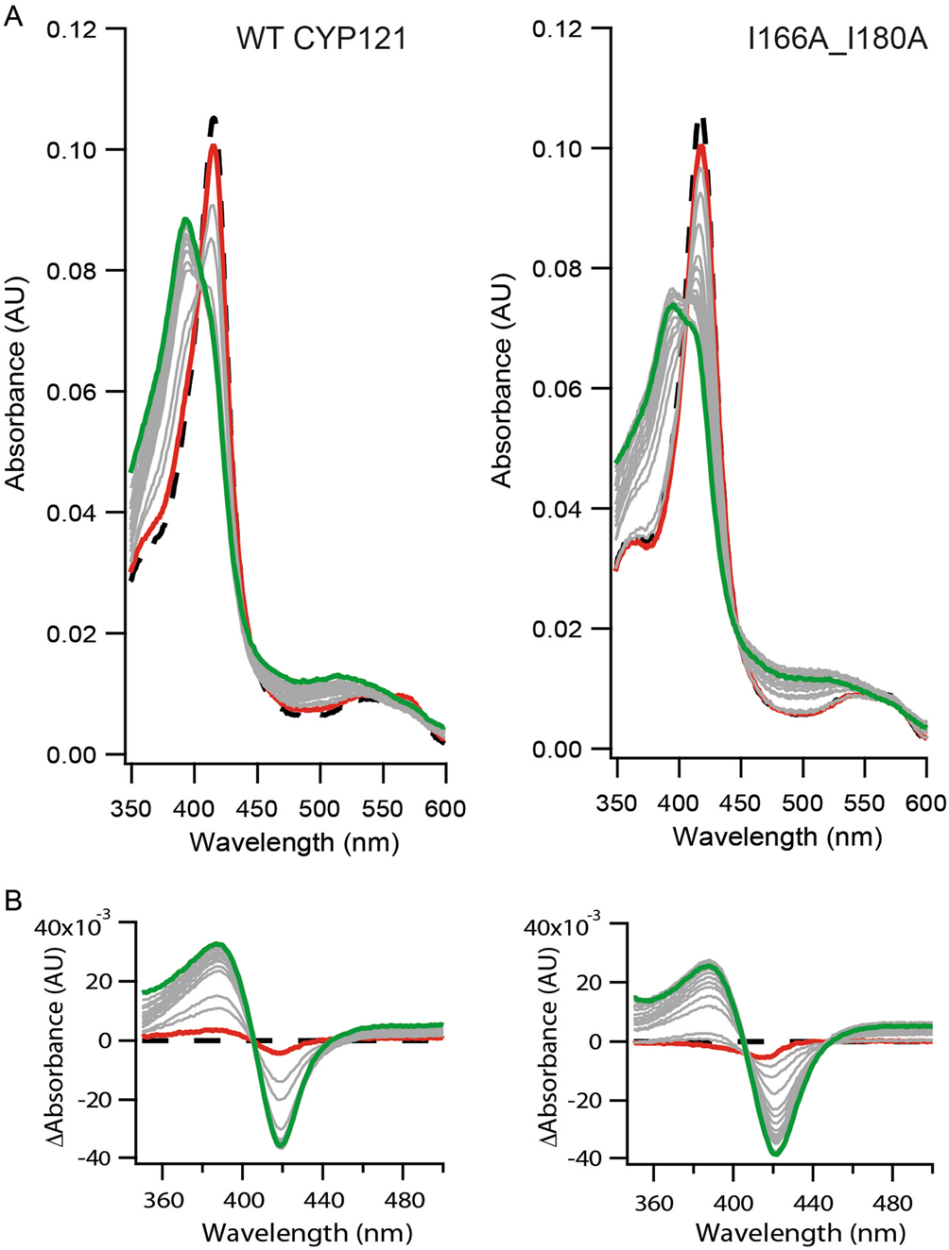
Spectral binding of cYY with WT and I166A_I180A CYP121A1. (**A**) cYY titration between 0 and 1050 µM produces a shift to 395 nm (green traces). (**B**) Subtracted spectra of cYY binding. Spectral binding plots were generated using Igor Pro Version 6.37 (https://www.wavemetrics.com/) and graphics designed by Adobe Illustrator CS5 (https://www.adobe.com/).

**Figure 4:**
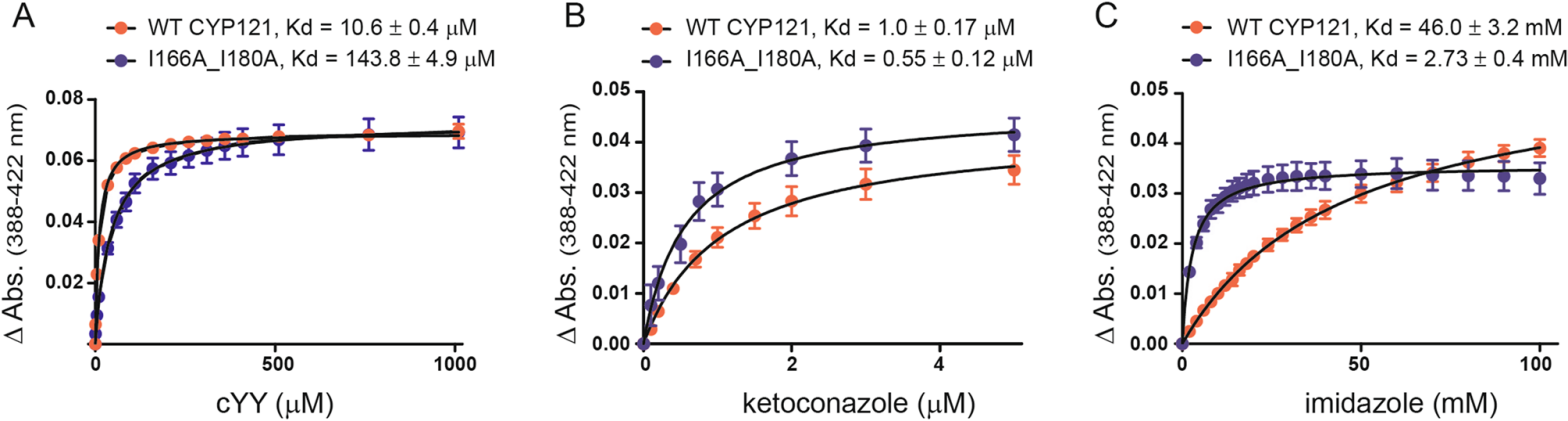
Ligand binding in WT CYP121A1 and I166A_I180A. Ligand titrations of cYY, ketoconazole, and imidazole in (**A**), (**B**), and (**C**), respectively. Binding curves were generated by plotting difference spectra against ligand concentration. Spectral binding plots were generated using GraphPad Prism 5 (https://www.graphpad.com/scientific-software/prism/) and graphics designed by Adobe Illustrator CS5 (https://www.adobe.com/).

The next phase of the investigation was to determine whether loss of functional dimerization results in disruption of binding to substrate and ligands in general. Purified I166A_I180A CYP121A1 produced a comparable absorption spectrum (dashed line in Fig. 3A, right panel) to that of WT, although a slightly narrower Soret band in the unliganded spectrum of the monomer was observed. Comparative spectral binding assays with the substrate and with two structurally dissimilar and non-specific azoles, imidazole, and the broad spectrum CYP inhibitor ketoconazole, were performed. Titration of cYY against monomeric CYP121A1 produced the predicted blue shift of the Soret peak from 418 nm to approximately 395 nm, as expected by substrate displacement of a weakly-bound active site water molecule^8,9^ (Fig. 3). However, when compared to dimeric CYP121A1, the monomer displays a weakened interaction with cYY by fourfold (Fig. 4A), along with a lower percentage of high spin population upon maximum binding. These data suggest that in the monomeric form, substrate access to the heme is at least partially disrupted.

The non-specific inhibitor ketoconazole does not significantly inhibit Mtb growth^10^ but was selected for this study as an additional ligand due to its large size disparity relative to cYY. Ketoconazole induced a red-shift in the Soret peak for both forms of CYP121A1 to 424 nm, as is typical for a type-II CYP ligand. However, in this case, an approximately twofold increase in affinity in monomeric CYP121A1 was observed, which was the opposite of the effect observed with cYY (Fig. 4B). A subsequent titration using imidazole as another non-specific but weaker-binding type-II ligand had a more significant effect, with Kd values decreasing 20-fold from 46 mM for dimeric CYP121A1 to 2.73 mM for the monomeric enzyme (Fig. 4C). These findings indicate that loss of dimerization in CYP121A1 inversely impacts ligand binding; substrate binding is negatively affected, while non-specific ligand binding is enhanced, thus indicating that the homodimer interfacial environment is a necessary component for the specific recognition of cYY.

### cYY stabilizes the heme thiolate-CO complex in monomeric CYP121A1

**Figure 5:**
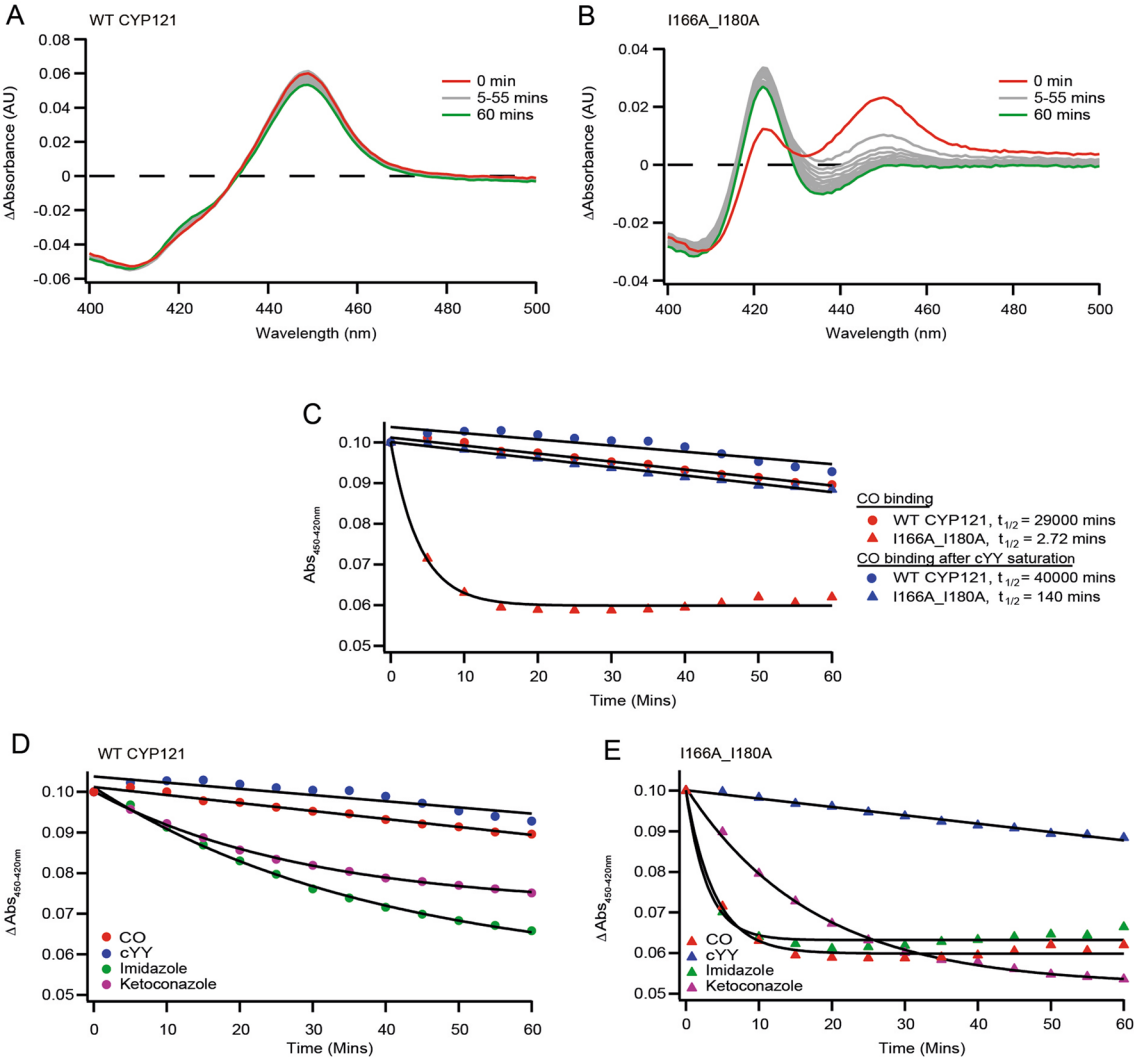
CO-bound spectra of WT CYP121A1 and I166A_I180A. The heme thiolate CO complex is stable in WT CYP121A1 (**A**) but rapidly converts to P420 in I166A_I180A (**B**). The CO-bound complex is stabilized for I166A_I180A in the presence of 1 mM cYY (**C**). Saturation with non-specific azoles imidazole and ketoconazole conferred limited stability to the P450 species (**D**,**E**). CO binding plots were generated using Igor Pro Version 6.37 (https://www.wavemetrics.com/) and graphics designed by Adobe Illustrator CS5 (https://www.adobe.com/).

In CYPs, reduction of the heme thiolate followed by complex formation with CO generates a characteristic Soret shift to 450 nm (P450)^11^. In this diagnostic assay, the heme thiolate-CO complex then undergoes a time-dependent conversion to the inactive form at 420 nm. The heme thiolate-CO complex in CYP121A1 is known to be pH sensitive, with protonation of the thiol leading to a reversible conversion to P420^12^. Time-dependent P450 to P420 conversion at a constant pH of 7.4 was measured in order to compare the stability of the heme thiolate-CO complex between monomeric and dimeric enzyme. Under saturating dithionite and CO conditions, the P450 species of wild-type, dimeric CYP121A1 was found to be very stable, persisting for close to 60 min (Fig. 5A). However, under the same conditions I166A_I180A (monomeric) CYP121A1 forms less of the CO-bound complex, which then also undergoes comparatively rapid conversion to P420 (Fig. 5B).

The kinetics of CO binding in CYPs are known to be influenced by the presence of substrate^13,14^. Therefore, the CO difference assay with CYP121A1 bound to cYY was performed. While little change in wild-type enzyme was observed, the estimated half-life of the heme thiolate-CO complex in monomeric CYP121A1 was significantly increased (by 50-fold) by the presence of the substrate (Fig. 5C). These findings are consistent with concurrent binding of CO with at least one molecule of cYY, resulting in substrate stabilization of the heme thiolate-CO complex. While this effect may also occur in dimeric CYP121A1, it may be more readily observed in the monomeric form due to the apparent instability of its substrate-free thiolate heme-CO complex.

To determine whether stabilization of the intact CO-bound complex in monomeric CYP121A1 was a result of general, non-specific ligand binding, or was specific to cYY, the CO difference assays in the presence of saturating concentrations of imidazole and ketoconazole were performed. While minimal stabilization of the 450 nm species was observed, the effect was far less than that observed with the substrate (Fig. 5D). Our interpretation of these data is that while disruption of the dimer interfacial environment negatively impacts the specificity of cYY binding, some substrate specificity appears to be retained in the monomer, as evidenced by the ability of cYY to rescue a stable substrate, CO complex.

### Thermal shift assays of WT CYP121A1 and I166A_I180A CYP121A1

**Figure 6:**
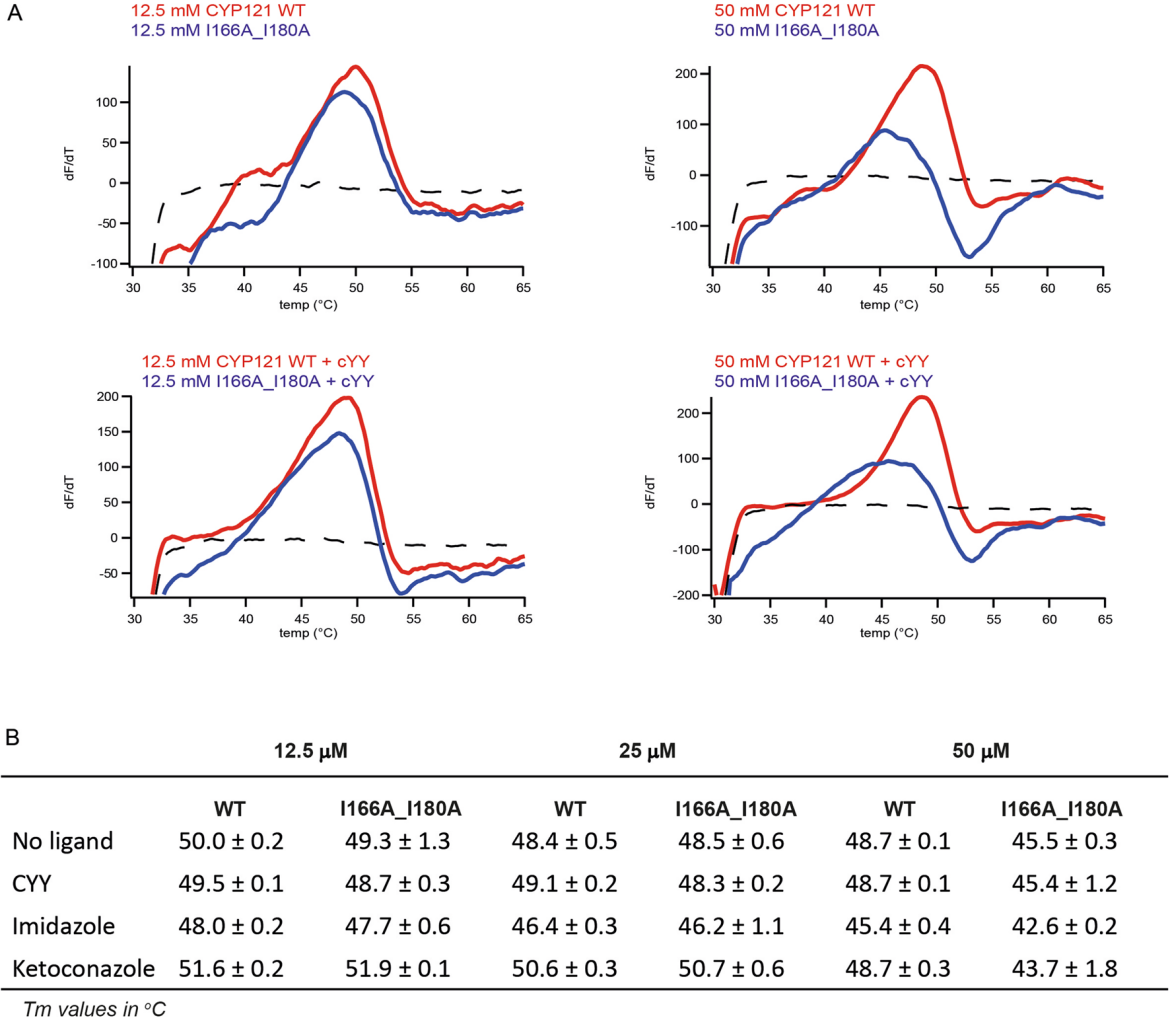
Tm calculations for WT CYP121A1 and I166A_I180A. (**A**) First derivative changes in fluorescence in response to temperatures (dF/dT) overlaid for both forms of the enzyme at two different concentrations (top panel) and again in the presence of 500 μM substrate (lower panel). Tm values at all concentrations and with all ligands tested are summarized in (**B**). Complete first derivative data for all experiments are included in Supplementary Fig. S5. Thermal traces in (**A**) were generated using Igor Pro Version 6.37 (https://www.wavemetrics.com/) and graphics designed by Adobe Illustrator CS5 (https://www.adobe.com/).

Structural destabilization resulting from loss of dimerization could also explain loss of CYP121A1 function in the monomeric form. To investigate this possibility, the GloMelt fluorescent dye from Biotium^15^ was used to monitor temperature-induced denaturing of CYP121A1. At a concentration of 12.5 μM, WT protein produced a change in fluorescence that was biphasic, with an inflection at approximately 39 °C and a second intense change at 50 ± 0.2 °C (Fig. 6A, red trace). In contrast, I166A_I180A produced only a single inflection at 49 ± 1.3 °C (Fig. 6A, blue trace). A calculated Tm of 50 °C is consistent with the thermal stability of CYP121A1 reported earlier^16^. These data suggest that at this concentration, which is most similar to the concentration of the enzyme in our functional studies, the monomeric enzyme has comparable structural stability to the dimeric form. Of note is the initial change in fluorescence of wild-type CYP121A1 at 39 °C, which is absent in the thermal scan of I166A_I180A, and therefore may represent dissociation of the dimer (thus exposing the non-polar dimer interface to the hydrophobic dye). Interestingly, at a higher concentration of 50 μM, this initial signal is greatly reduced in wild-type CYP121A1. Since it is unlikely that the protein becomes monomeric as concentration increases, this change was interpreted to mean that CYP121A1 dimers experience enhanced thermal stability at higher concentrations, and therefore dimer dissociation and denaturing of the CYP fold are concurrent events measured as a single change in fluorescence, which is also consistent with a decrease in the apparent Tm of WT protein by one degree at 50 μM (Fig. 6B). In contrast, the Tm of I166A_I180A decreased by several degrees when the concentration is increased. The reasons for destabilization of the monomeric enzyme at these concentrations are likely multifactorial, but may include heat-induced aggregation at high concentrations or excluded volume induced by molecular crowding.

As a way to determine whether ligand contributes to the stability of CYP121A1 monomers and dimers, the Tm data was re-acquired in the presence of cYY and the non-specific ligands imidazole and ketoconazole (Fig. 6B). Here it was observed that the substrate did not significantly alter the Tm of either wild-type protein or I166A_I180A. The addition of ketoconazole caused a modest increase in the stability for both forms at lower protein concentrations. However, for reasons that are not yet clear, ketoconazole and imidazole were both destabilizing to I166A_I180A at 50 μM. An interesting observation of the ligand-bound wild-type enzyme was that the low-temperature change in fluorescence, which we believe represents dissociation of the dimer, was not present at all for any of the concentrations measured (Fig. 6A, Supplementary Fig. S5). This effect may be due to ligand-induced stabilizing of CYP121A1 dimers, as may occur at high protein concentrations. However, the possibility that at a concentration of 12.5 μM, CYP121A1 becomes a monomer upon ligand binding cannot be ruled out. This aspect of CYP121A1 dimerization warrants further investigation.

### The CYP121A1 dimer interface influences the environment at the FG-loop

Two of the surface residues that promote dimer formation in CYP121A1 are located on the adjacent F and G α-helices. It is well established that remodeling of the distal surface of CYPs at the F and G helices correlates with the opening of specific substrate access channels toward the active site^17–19^. For example, in the camphor-metabolizing enzyme P450cam, repositioning of the flexible intervening FG-loop is required to establish the open conformation of the enzyme^19^. In order to understand how the dimer interface of CYP121A1 affects the local structure at this site, ^19^F-NMR was used as a means to monitor the FG loop in both the monomer and dimer of the enzyme. Fluorine nuclei are naturally NMR observable (spin of ½), are highly sensitive to changes in the electrochemical environment, and can be incorporated in a site-specific manner^20,21^. Moreover, the sensitivity of the fluorine nucleus is particularly useful as a structural probe for a dimer complex that, due to its size, is otherwise inaccessible by a ^15^N labeling strategy.

**Figure 7:**
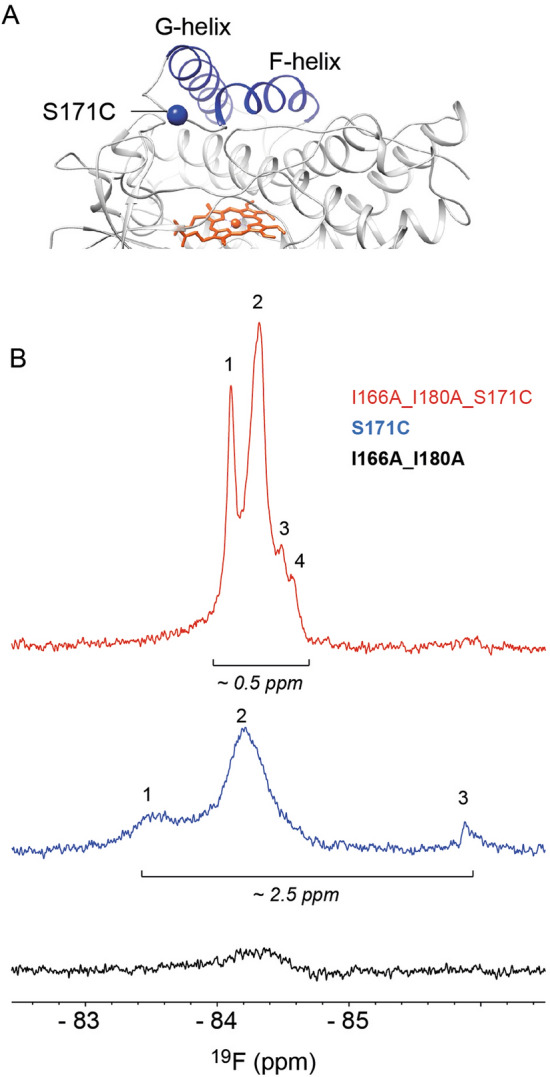
^19^F-NMR of the FG-loop of CYP121A1. (**A**) The positioning of the cysteine mutation between the F and G α-helices. (**B**) 1D spectra of the FG-loop in CYP121A1 monomer (top trace), dimer (middle trace), and monomer without the S171C mutation (bottom trace). All spectra are scaled equally. The figure in (**A**) was generated using UCSF Chimera v1.14 (www.cgl.ucsf.edu/chimera). Spectra in (**B**) were generated using TopSpin Version 4.0.6 (https://www.bruker.com/).

Here we incorporated a cysteine mutation at residue Ser-171 due to its position near the predicted homodimer interface at the center of the intervening loop between the F and G α-helices (Fig. 7A). Incubation of the cysteine mutant with the thiol-reactive compound BTFA results in the incorporation of a trifluoromethyl side chain in place of Ser-171. One-dimensional spectra of labeled S171C (dimeric form), I166A_I180A_S171C (monomeric form), and I166A_I180A (monomeric, without the cysteine mutation) were acquired at matching protein concentrations and acquisition parameters (Fig. 7B).

CYP121A1 contains three native cysteine residues. Of these, Cys-345 represents the axial coordinating ligand of the heme iron and therefore was not expected to react with BTFA. Two more residues, Cys-51 and Cys-147, are located in α-helical environments and were therefore expected to produce broad ^19^F-NMR spectra relative to the non-native cysteine residue in the unstructured FG-loop. This is supported by a comparison of the broad spectrum of the I166A_I180 mutant (bottom trace) with spectra of CYP121A1 containing the S171C mutation. From these experiments, the observable resonance signals in the top two traces of Fig. 3B were assigned as S171C of the FG-loop.

From this study, it was observed that the FG-loop in dimeric CYP121A1 (center trace) is exchanging between one primary conformation, labeled as resonance 2 in Fig. 3B, and two sub-conformations (resonances 1 and 3). The detection of distinct, dispersed signals indicates that the FG-loop occupies a structured environment in the CYP121A1 dimer. In contrast, monomeric CYP121A1 (top trace) contains sharper, narrow resonances with four resolvable sub-conformations of the FG-loop. Notably, the net dispersion of all of the signals is reduced, from 2.5 ppm in the dimer to 0.5 ppm in the monomer. This is consistent with the increased disorder at the FG-loop when the second molecule of the dimer is removed. The relative proportions of peaks 1 and 2 in the dimer appear was also noted to be preserved in peaks 1 and 2 of the monomer. This was not expected since maximum disorder at the FG-loop would appear as a single sharp resonance. This most likely indicates that, while the disorder of the FG-loop has increased, some of the conformational heterogeneity at the site is due to intramolecular interactions between the FG-helices and the rest of the protein, as might be expected from slowly exchanging open and closed conformations of the enzyme.

### Modeling the CYP121A1 dimer

The functional homodimer interface of CYP121A1 described in this study is not represented in the crystallographic record. Therefore, the dimer was modeled using the docking program HADDOCK 2.2 (http://milou.science.uu.nl/services/HADDOCK2.2)^22^. The non-polar residues identified previously (Ile-166, Ile-180, and Val-379) were entered as active interaction constraints. In order to avoid introducing an orientation bias, no further input was provided regarding the specific pairing of intermolecular contacts.

**Figure 8:**
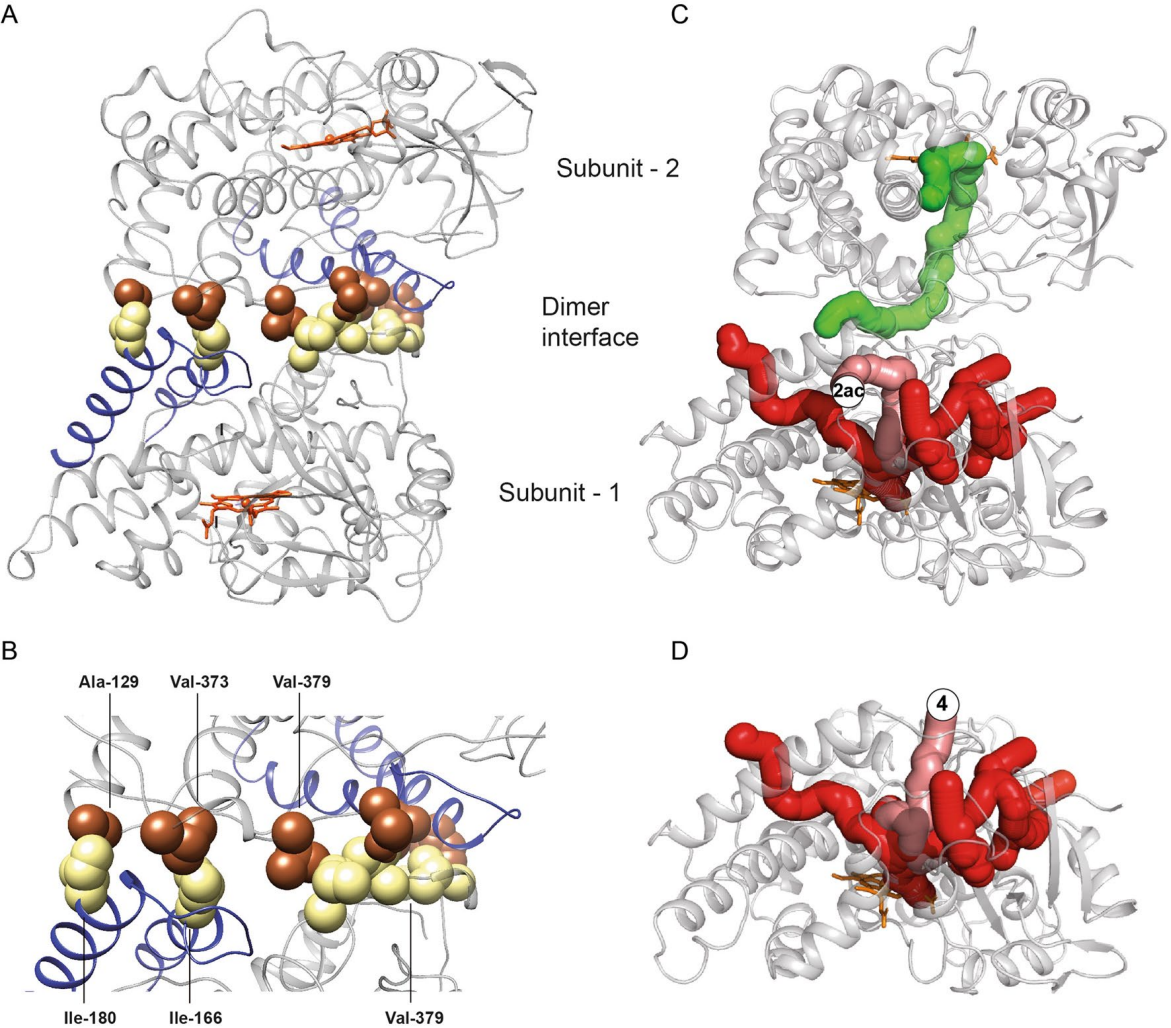
Docked model of the functional CYP121A1 dimer and ligand access in the monomeric and dimeric enzyme. (**A**) Subunits are oriented in a distal-to-distal arrangement with the FG-loop (blue) positioned on opposite sides of the complex. The hydrophobic side chains (**B**) are highlighted in yellow and rust colors for different subunits and interact in an interlocking arrangement. Available ligand access channels were analyzed for a CYP121A1 dimer (**C**) and for CYP121A1 monomer (**D**). The salmon-colored tunnel on subunit-1 was differentially positioned due to the dimer interface. The only solvent channel found to extend between the subunits is highlighted in green. Structure figures were generated using UCSF Chimera v1.14 (www.cgl.ucsf.edu/chimera) and PyMOL version 2.3.4 (https://pymol.org/2/).

The modeled complexes were categorized by similarity into nine primary clusters (see Supplementary Fig. S6). The solutions were evaluated based on energy minima calculated from van der Waals and electrostatic interactions, in addition to the degree of similarity [interfacial root-mean-square-deviation (i-RMSD)] to the monomer structure (PDB ID 3G5F) used as both receptor and ligand inputs. In general, solution clusters grouped into two tiers; clusters in which the F and G α-helices from different molecules interact directly scored poorly, with i-RMSD values above 10 Å, while clusters in which the F and G α-helices are arranged on opposite sides of the dimer scored significantly better, with i-RMSD values between < 1 and 3 Å. The most stable orientation of the CYP121A1 dimer, shown in Fig. 8A, was found to be consistent with favorable models produced by the independent docking program ClusPro^23^.

The modeled dimer reflects a 180° superposition of CYP121A1 monomers about a plane composed of the F and G α-helices and the β3-loop, with each heme plane in near-parallel alignment. The interface has a calculated area of approximately 1300 Å^3^. The FG-loop regions are oriented in the same direction but on opposite sides of the complex. The non-polar side chains, highlighted in yellow and rust color (Fig. 8B), interact in an interdigitate arrangement at the interface that forms a semi-crescent when the dimer is viewed lengthwise. The model also revealed that additional, previously unidentified side chains might participate in intermolecular hydrophobic contacts. For example, Ala-129 and Val-373 of one CYP121A1 subunit interact with Ile-166 and Ile-180 of a second subunit. Consistent with the ^19^F-NMR spectra indicating that the FG-loop occupies an ordered environment, the dimer model reflects the FG-loop located between the proline-rich BC-loop of the same molecule and the β3-loop of the second molecule.

Since the CYP121A1 monomer and dimer differentially bind ligands, how the dimer orientation impacts potential substrate access channels was investigated. The modeled dimer was analyzed using the ChannelsDB server (https://webchemdev.ncbr.muni.cz/ChannelsDB/)^24^, and the results compared using the same parameters for the monomer structure (Fig. 8C,D). From this analysis, it was observed that most solvent channels that are large enough to accommodate cYY and are oriented parallel to the plane of the heme do not appear to be affected by the dimer interface. However, channels that would otherwise be oriented perpendicular to the heme appear to be obstructed by the second molecule of CYP121A1. The channel designated as channel **4** in CYP nomenclature^17^ that allows access to the active site between the FG-loop is instead redirected into channel **2ac** in the dimer, located between the G-helix and the BC-loop. However, one potential solvent channel in CYP121A1 dimers was detected (green channel, Fig. 8C) that diverges from channel **4** and extends through the F and G α-helices towards the active site cavity of the second molecule. Therefore, the possibility of ligand exchange between the two dimer active sites cannot be excluded.

## Discussion

Although its role in Mtb biology remains to be fully determined, CYP121A1 appears to be a viable target in the development of anti-tuberculosis agents. This is due in part to the essentiality of the enzyme, the uncommon nature of the phenol-coupling reaction, and the correlation between azole minimal inhibitory concentrations and their corresponding affinity for CYP121A1^3,25^. However, our current understanding of CYP121A1 function does not encapsulate contributions, if any, from dimer formation. Moreover, recent ligand docking and molecular dynamics simulations do not account for contributions from the second molecule of CYP121A1^26,27^. In this work, the CYP121A1 dimer has been disrupted by introducing alanine substitutions of two isoleucine residues on the distal surface (I166A_I180A). Monomerization of the enzyme is reported by size exclusion chromatography (Fig. 1) as well as by membrane filtration of the mutant proteins (Supplementary Fig. S4).

The engineered monomer displays a marked decrease in catalytic activity toward its tyrosine dipeptide (cYY) substrate. A combination of ligand binding experiments and thermal shift assays suggest that loss of function is due in part to the loss of specificity for cYY, rather than destabilization of the CYP fold. Notably, ligand binding was not uniformly affected; cYY binding was compromised in the absence of the dimer, while the affinity for non-specific azoles increased (Fig. 4). Notably, the approximate 75% reduction in catalysis correlates with a fourfold loss of affinity for cYY, suggests that compromised substrate binding is a significant cause of decreased enzyme function. However, while substrate binding was compromised, not all specificity was lost in the monomer, as suggested by the ability of cYY to stabilize the heme-thiolate CO complex in I166A_I180A (Fig. 5). This innate specificity may be related to the observation that the FG-loop in monomeric CYP121A1, while more disordered, nonetheless retains some of its heterogeneity (Fig. 7), likely due to intramolecular interactions. The FG-loop factors prominently in determining substrate binding in other CYPs^28,29^ and the substrate-bound crystal structure of CYP121A1 reflects cYY bound near the FG-loop via stabilizing π-stacking interactions with residue Phe-168^4,30^. Therefore, cYY specificity may well be a product of both specific active site interactions as well as dimer interface stabilization of the FG region. A more comprehensive study of ligand-induced perturbation of the FG-loop is clearly warranted.

Curiously, none of the reported CYP121A1 structures reflect a distal-to-distal orientation in the crystal lattice. CYP121A1 crystallizes primarily as a monomer, with the exception of structures of fluconazole-bound CYP121A1, which reflect a side-to-side packing interface nearly perpendicular to the one reported here^6^. An apt comparison may be the ligand-induced crystallographic dimer that has been described previously for CYP130 of Mtb, which is also influenced by hydrophobic contacts and involves the G-helix, the BC-loop, and the N-terminal end of the I-helix^31^. However, the CYP121A1 interface is distinctive in important ways; the interfacial surface area is comparatively smaller than CYP130 (~ 2000 Å^3^ in CYP130 compared to 1300 Å^3^ for CYP121A1), and dimer formation at higher protein concentration is not affected by ligand binding (Supplementary Fig. S1). We now also theorize that the ligand-induced disruption of CYP121A1 dimers, as determined by ionization mass spectrometry, may have been due to a disproportionate ligand-to-protein ratio (20-fold higher), thus resulting in non-specific surface disruption of the dimer interface^5^.

Therefore, to our knowledge, the particular dimer interface in CYP121A1 has not previously been described in bacterial CYPs. Modeling of the complex using the hydrophobic residues as non-biased restraints resulted primarily in two clusters of solutions (Supplementary Fig. S6), in which the discriminating factor was the orientation of the monomers; positioning the FG-loop region on opposites sides of the complex was energetically favored while positioning the FG-loops together on the same side was disfavored. Functionally, it is clear that CYP121A1 relies on a dimer arrangement for stabilization of the active complex (see CO binding data in Fig. 5). Therefore, destabilization of the CO-bound complex in monomeric enzymes may be due to perturbations within the active site. One example is the active site residue Arg-386, which has a demonstrated role in determining redox potential in CYP121A1 and may act as a proton relay in catalysis^3,32^. In our model, the Arg-386 backbone is located adjacent to the dimer interface due to its proximity to a hydrophobic contact at residue Val-379 and, therefore, maybe in a position to be affected by changes in the FG-loop of the neighboring CYP121A1 molecule. Outside of the active site cavity, the quaternary distal-to-distal arrangement of CYP121A1 is also likely to be functionally useful, as the opposite orientation of the CYP121A1 molecules would allow substrate binding to be modulated at two different sites, while also allowing unimpeded interactions with cognate Mtb redox partners on the proximal surface. Interestingly, the arrangement of a CYP121A1 molecule in the dimer mirrors the arrangement of a mammalian CYP in a membrane bilayer, where interactions between the FG-loop and phospholipids help regulate substrate binding, rather than a dimer interface. Sequence alignment of Mtb CYPs or between CYP121A1 with other bacterial CYPs does not suggest that surface hydrophobics along the FG-region are highly conserved. However, it is nevertheless possible that functional dimerization relies on interactions besides non-polar contacts, and that the dimer interface described here may occur in other bacterial enzymes as well. Last, we should also note that while the solution dimer interface is not directly represented in crystal structures, we believe that the dimer orientation, as modeled here, may exert some influence on the co-crystallization of ligands. For example, an overlay of ligand-bound CYP121A1 structures indicates that the likely access channel that connects the active site with the protein surface forms nearly parallel to the heme plane between the I-helix and the G-helix (channel 2c), consistent with dimer interface redirection to the adjacent channel 2ac (Fig. 8C,D), while other access routes that are perpendicular to the heme are obstructed.

In summary, this work describes a functional dimeric interface for an essential enzyme in Mtb. Dimerization of CYP121A1 contributes to specific substrate binding in vitro, with a loss of function when dimers are disrupted. Although the significance of enzyme dimers for in vivo function remains an open question, the observation that the apparent specificity of substrate binding is imparted by the dimer interface should inform the design of potential CYP121A1 inhibitors.

## Materials and methods

### Protein expression and purification

The plasmid containing the CYP121A1 gene in a pET11a expression vector was a gift from the lab of Dr. Andrew Munro. This plasmid was modified (GenScript; https://www.genscript.com/) to contain a thrombin-cleavable N-terminal 6 × histidine tag. Expression and purification of WT and mutant CYP121A1 is described further under Supplementary data. Expression and purification of bovine adrenodoxin reductase and bovine adrenodoxin used in the activity assays were carried out as described previously^33,34^.

### Gel Filtration for CYP121A1 size determination

Purified WT CYP121A1 and CYP121A1 containing single or double surface mutations were concentrated to 50 μM prior to loading onto a calibrated and pre-equilibrated 24 ml Enrich 650 gel filtration column (Bio-Rad; https://www.bio-rad.com/). The running buffer consisted of 50 mM Tris–HCl, 300 mM NaCl, pH 7.4. The column was run at a flow rate of 0.5 ml/min, and CYP121A1 was monitored using wavelengths of 280 nm and 420 nm. General calculations of molecular weights from gel filtration were confirmed by passage of the purified WT and mutant enzyme in gel filtration buffer through a 50 kDa spin filter (10,000*g*) followed by absorption quantification of protein in the flow-through fractions (Supplementary Fig. S4).

### Spectral binding assays

Substrate and inhibitors binding was assayed in triplicate using ligand-induced changes in the UV–Vis absorption spectra of CYP121A1. Briefly, cYY (contributed by the Simons lab) and ketoconazole were titrated from a DMSO stock into a 1 µM solution of CYP121A1 in 50 mM TrisHCl, 300 mM NaCl, pH 7.4. Imidazole titrations were added from an aqueous stock. Spectra were acquired at ambient temperature from samples in a 1 cm quartz cuvette on a dual-beam Shimdazu 2700 spectrophotometer (https://www.ssi.shimadzu.com/). To allow for complete binding, each ligand addition was followed by a 10 min incubation period prior to the acquisition of the spectrum. Affinity constants were calculated by fitting the ligand concentrations plotted against the difference spectra showing either a red-shifted, type-I response (cYY) or a blue-shifted, type-II response (ketoconazole and imidazole). Data fitting was carried out using Prism GraphPad v7.05 using a single binding mode equation for hyperbolic binding as described previously^33^. Carbon monoxide (CO) binding spectra were performed by first reducing samples of CYP121A1 with excess sodium dithionite, followed by bubbling of CO gas and immediately recording spectra between 250 and 700 nm at regular intervals of one scan every 5–10 min for one hour. The difference spectra were plotted by subtracting CO bound spectra from the reduced spectra. The rate of loss of the CO-bound signal at 450 nm was calculated based on a plot of the conversion to 420 nm over time. To evaluate the ligand-induced effects on the stability of the CO-heme complex, a time-course CO binding assay was performed upon completion of ligand spectral binding assays.

### Reconstitution CYP121A1 assay

A CYP121A1 functional assay was reconstituted using adrenodoxin (Adx) and adrenodoxin reductase (Adr) from bovine. The assay contained active CYP121A1 (5 µM as determined by the initial CO-bound peak at 450 nm), Adx and Adr at 15 µM each, and cYY (150 µM) in 50 mM TrisHCl (pH 7.4). The reaction mixture was preincubated for 5 min at 30 °C, and the reaction was initiated upon addition of 1 mM NADPH. The reactions were quenched after 0, 15, 30, and 60 min by the addition of 22.2 µl of 20% nitric acid in 200 µl reaction mixture. Tryptophan (5 μM) was added as an internal standard. 50 μl of the quenched reaction was removed and combined with 50 μl of 100% acetonitrile, followed by high-speed centrifugation 10,000 rpm for 30 min. 20 μl of the supernatant was diluted tenfold into a mobile phase solution consisting of 10% acetonitrile and 0.1% formic acid in water. Samples were resolved on a Poroshell 120 EC-C18 column (4.6 mm × 250 mm) (Agilent; https://www.agilent.com/) using a 30 min isocratic mobile phase run on an Agilent 1260 Infinity II liquid chromatography system. Elution peaks were monitored at wavelengths of 214 nm and 278 nm. Substrate depletion was calculated by comparing the ratio of the peak area for the cYY elution (15.5 min) with the area under the tryptophan elution peak (12.5 min). All data were compiled from replicates of five reactions.

### Glutaraldehyde cross-linking

Chemical cross-linking of CYP121A1 was carried out using 200 µl aliquots of 30 µM protein in 20 mM HEPES buffer, pH 8.2. CYP121A1 was saturated with econazole for added stability. The cross-linking reaction was initiated by the addition of 10 µl of a freshly prepared solution of 2.3% glutaraldehyde. The reaction was stopped after 20 min by the addition of 80 µl of a 1 M TrisHCl solution. Cross-linked dimers were resolved by electrophoresis on a 5–12% gradient acrylamide gel. Reactions were run in triplicate and subjected to chymotrypsin digest and mass spectrometric analysis as liquid samples. A sample of CYP121A1 that had not been treated with glutaraldehyde was used as a reference.

### Chymotrypsin digest and LC–MS/MS

Each in-solution sample was denatured in 150 µl of ice-cold lysis buffer (50 mM Tris-formic acid, 150 mM NaCl, 0.5% sodium deoxycholate, 2% SDS, 2% NP-40, pH 8.0). An acetone precipitation/on-pellet-digestion procedure was applied to perform precipitation and tryptic digestion for reproducible peptide recovery, as described previously^35,36^.

The peptide mixture was separated and analyzed using an UltiMate 3000 RSLCnano-LC system coupled with an Orbitrap Fusion Lumos mass spectrometer. The mobile phase A contains 2% acetonitrile in 0.1% formic acid, and mobile phase B contains 88% acetonitrile in 0.1% formic acid. 4 µl of digested samples were loaded onto a large-ID trap (300 µm ID × 0.5 cm, packed with Zorbax 5-µm C18 particles) with 1% B at a flow rate of 10 µl/min for 3 min. The trapped peptides were then back-flushed onto the nano-LC column heated at 52 °C (75 µm ID × 60 cm, packed with Waters XSelect CSH 2.5-µm C18 particles) at a flow rate of 250 nl/min.

Mass spectrometry data were acquired under data-dependent and positive ion scan mode with a spray voltage of 2000 V, ion transfer tube temperature of 250 °C, and a cycle time of 3 s. One scan cycle included a survey scan (*m/z* 400–1500) at a resolution of 120,000 with an AGC target of 4 × 10^5^ and a maximum injection time of 50 ms. MS2 was performed by an isolation window of 1.2 Th with the quadrupole for high energy collision dissociation (HCD) fragmentation and detected by Orbitrap at a resolution of 15,000 with an AGC target of 5 × 10^4^. The maximum injection time was 50 ms, and the collision energy was 30%. Dynamic exclusion was enabled with a repeat count of 1 and an exclusion duration of 45 s.

The mass spectrometry raw files were searched against CYP121A1 protein sequence and its background of *Escherichia coli* database from UniProt using the Proteome Discoverer v1.4 (https://www.thermofisher.com/). The false discovery rate was determined by using a target-decoy search strategy. Scaffold 4.5 (Proteome Software, Portland, OR; http://www.proteomesoftware.com/) was used to validate MS/MS-based peptide and protein identification. The false discovery rates of 0.1% at the peptide level, a minimum of two unique peptides, and only 1 decoy protein were applied in this study. An ion current-based quantification method was used as previously described^37,38^. Thresholds of 2.0-fold change and a p-value cutoff of 0.05 were used to define the altered peptides. To determine the crosslinking sites between the glutaraldehyde-treated and control group, a minimum of two unique peptides were required to support the alteration.

### Thermal shift assays

GloMelt from Biotium was used for thermal shift assays using the standard protocol with slight modifications^15^. Proteins were prepared at different concentrations (12.5 µM, 25.0 µM, and 50 µM) in a buffer of 50 mM TrisHCl, 300 mM NaCl, pH 7.4. Samples were run in triplicate using 20 μl aliquots containing 1× GloMelt dye on a qPCR plate and sealed using BioRad Microseal^R^ ‘B’ optical seal. Samples were centrifuged on a PCR plate spinner prior to running on a Bio-Rad CFX96 Touch real-time PCR detection system. Denaturation was carried out over a temperature range between 20 to 99 °C with an increment of 0.2 °C and ramping of 0.01 °C/s.

Tm calculations were determined using the slope of the first derivative of the fluorescence curve, as analyzed by the Bio-Rad CFX Maestro Software, v3.1 (https://www.bio-rad.com/). For similar assays performed in the presence of ligands, cYY, imidazole, and ketoconazole were included at concentrations of 500 µM, 100 mM, and 20 µM, respectively.

### ^*19*^*F-NMR spectroscopy*

CYP121A1 eluted from the Ni–NTA column was diluted to 2 µM in 50 mM TrisHCl, pH 7.4, 300 mM NaCl, and supplemented with 10 mM 3-bromo-1,1,1-trifluoroacetone (BTFA) and 5 mM dithiothreitol (DTT). Protein was incubated overnight at 4 °C without agitation, after which the unreacted BTFA and DTT were removed by gel filtration on a 120 ml bed volume column. 125 nmol of BTFA labeled protein was exchanged into NMR buffer (50 mM potassium phosphate, pH 7.4, 50 mM NaCl, and 10% D_2_O) using a 10-kDa molecular mass cut-off filter and adjusted to a final concentration of 250 µM. ^19^F-NMR spectra were acquired at 25 °C on an Agilent 400 MHz spectrometer using a 30° pulse angle, a 1 s delay, and − 84 ppm transmitter offset frequency for 10,000 scans. Raw data were processed and analyzed using Bruker, TopSpin version 4.0.6 (https://www.bruker.com/).

### HADDOCK Docking of CYP121A1 dimer

Protein docking was carried out using the HADDOCK 2.2 server (http://milou.science.uu.nl/services/HADDOCK2.2)^22^. Ile-166, Ile-180, and Val-379 were used as general, non-biased interaction constraints between monomers of a CYP121A1 structure (PDB ID 3G5H)^4^. Modeled solution clusters of dimeric CYP121A1 were evaluated based on minimal interfacial root mean square deviation and optimal Van der Waals and electrostatic energy calculations. The area occupied by the dimer interface was calculated using PDBePISA (https://www.ebi.ac.uk/pdbe/pisa/)^39^, and solvent access channels were determined using the ChannelsDB database server (https://webchemdev.ncbr.muni.cz/ChannelsDB/)^24^.

## Supplementary Information

Supplementary Information.
