# Supplementary material for: Surface hydrophobics mediate functional dimerization of CYP121A1 of Mycobacterium tuberculosis
Source: Sci Rep. 2021 Jan 11;11:394. doi: 10.1038/s41598-020-79545-y (PMC7801616; doi:10.1038/s41598-020-79545-y)
Supplement: Supplementary file 1 — Supplementary Information. [file 41598_2020_79545_MOESM1_ESM.docx]

**Supplementary Data:**

**Working title:**

Surface hydrophobics mediate functional dimerization of CYP121A1 of *Mycobacterium tuberculosis*

Amit Kumar^1^, Christopher S. Campomizzi^1^, Natalie Jay^1^, Shaun Ferguson^1^, Emelie-Jo Scheffler^1^, James Lioi^1^, Chengjian Tu^2^, Jun Qu^2^, Claire Simons^3^ and D. Fernando Estrada^1^*

1 **Department of Biochemistry, Jacobs School of Medicine and Biomedical Science, University at Buffalo, Buffalo, New York -14203, United States**

**2 Department of Pharmaceutical Sciences, School of Pharmacy, University at Buffalo, Buffalo, New York 14214, United States**

**3 School of Pharmacy & Pharmaceutical Sciences, Cardiff University, King Edward VII Avenue, Cardiff, CF10 3NB U.K.**

**Materials and Methods**

*Expression and purification of WT and mutant CYP121A1*

C41 BL21 or JM109 competent cells were transfected with the expression plasmid. A single colony was used for a 5 ml starter culture in Luria Broth (LB), followed by an overnight culture in 100 ml LB, and scaling up to one liter in Terrific Broth media. All growth cultures were supplemented with 50 µg/ml carbenicillin. Expression was carried out with shaking at 200 rpm at 37 °C until cells reached an optical density of 0.8 at 600 nm. Cells were induced by the addition of 250 mg/liter isopropyl β-D-1-thiogalactopyranoside and 80 mg/liter δ-aminolevulinic acid, followed by an expression period of 48 hours with 180 rpm shaking at 22 °C. Cells were harvested by centrifugation at 4,500 x g for 20 minutes at 4 °C. Cell pellets were suspended in chilled 50 mM TrisHCl, 300 mM NaCl, pH 7.4, and stored at -80°C for future use.

Cells were thawed at ambient temperature and chemically lysed using lysozyme (1 mg per 3 ml cell lysate) at 4°C with gentle stirring. After 30 minutes, 1x Halt protease inhibitor mixture 1 uM PMSF, and 1μM DNase were added, followed by an additional 30 minutes incubation. The chemically lysed cells were mechanically lysed by sonication at 30 s intervals for a total of 3 mins. The soluble protein mixture was separated from cell debris by centrifugation at 100,000 x g for 30 minutes. CYP121A1 was captured by passing the supernatant through charged Ni-NTA resin pre-equilibrated with 50 mM TrisHCl and 300 mM NaCl, pH 7.4. Bound CYP121A1 was washed with loading buffer containing 50 mM imidazole and then eluted with the same buffer containing 100 mM imidazole. Eluent fractions were selectively pooled based on an optimal *Rz* purity ratio (A_418_/A_280_) above 2.0 and concentrated prior to passing through a calibrated 120 ml bed volume gel filtration column and using 50 mM TrisHCl and 300 mM NaCl, pH 7.4, as a running buffer. Removal of the N-terminal 6 x histidine tag was achieved by thrombin cleavage (1 unit of protease per mg of CYP121A1, with gentle agitation overnight at ambient temperature) followed by passing the digestion mixture through a charged Ni-NTA resin. The flow-through was then concentrated and cleaned of the affinity tag by passing through a gel filtration column for a second time. CYP121A1 containing surface dimer-disrupting mutations was purified using the same protocol. The final quantification of CYP121A1 was calculated using an iron extinction coefficient of 0.1 μM^-1^ cm^-1^ at 417 nm.

**
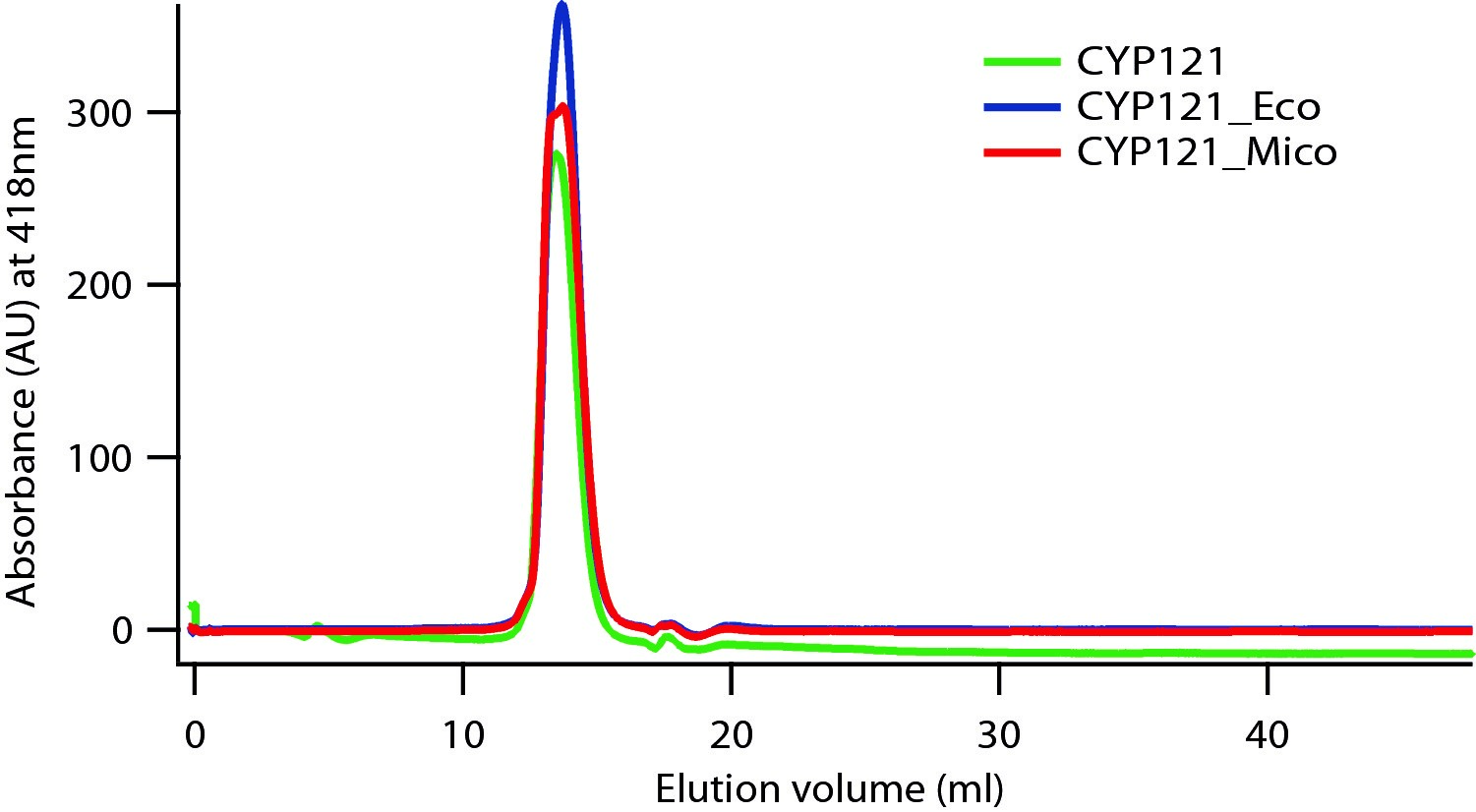
**

**Figure S1**: **Gel filtration chromatography of WT ligand-free and azole-bound CYP121A1.** The WT enzyme (50 μM) was pre-saturated with econazole (blue trace), miconazole (red trace), or run without ligand (green trace). Ligand binding was verified by a complete shift to 424 nm of the Soret band. Chromatographic traces were generated using Igor Pro Version 6.37 (https://www.wavemetrics.com/) and graphics designed by Adobe Illustrator CS5 (https://www.adobe.com/).

**Table S1.** **Peptide mapping of the CYP121A1 dimer interface.** Glutaraldehyde cross-linking produces peptides that are detected at different abundances in treated relative to untreated samples. Each peptide was normalized against the sum intensities of peptides without Lys. Peptides with an intensity ratio > 2 and with a p < 0.05 were determined to have higher intensity in untreated samples than those treated with the cross-linker. The modifications *m*, *n*, and *c* refer to methionine oxidation, asparagine deamidation, and cysteine carbamidomethylation, respectively.


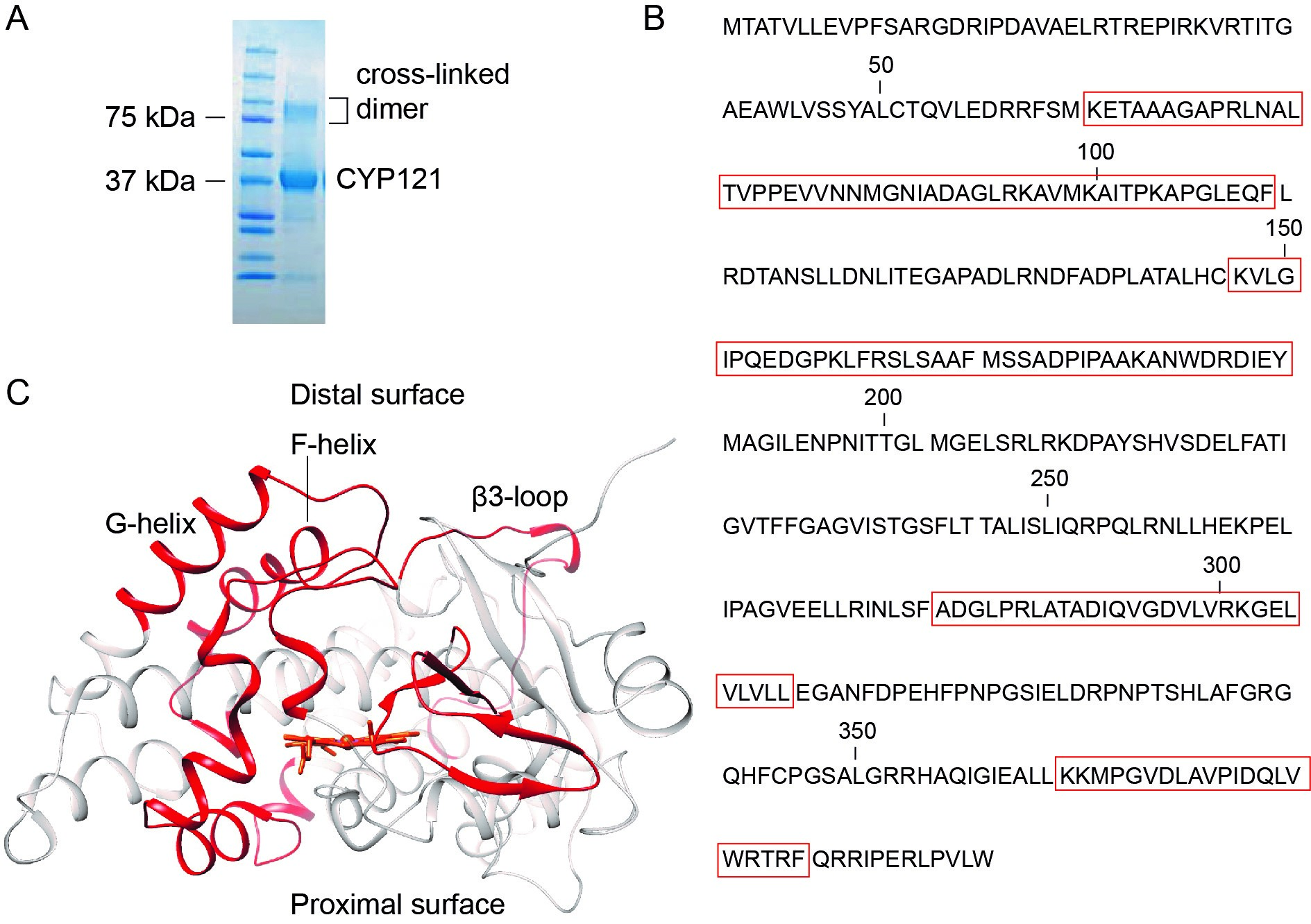


**Figure S2. Glutaraldehyde cross-linking and LC-MS/MS analysis of CYP121A1.** A) Denaturing gel electrophoresis of glutaraldehyde-treated CYP121A1. Modified peptides are indicated in red in the primary sequence in B) and mapped in the CYP121A1 structure in C). The structure figure in C) was generated using UCSF Chimera v1.14 (www.cgl.ucsf.edu/chimera).

**
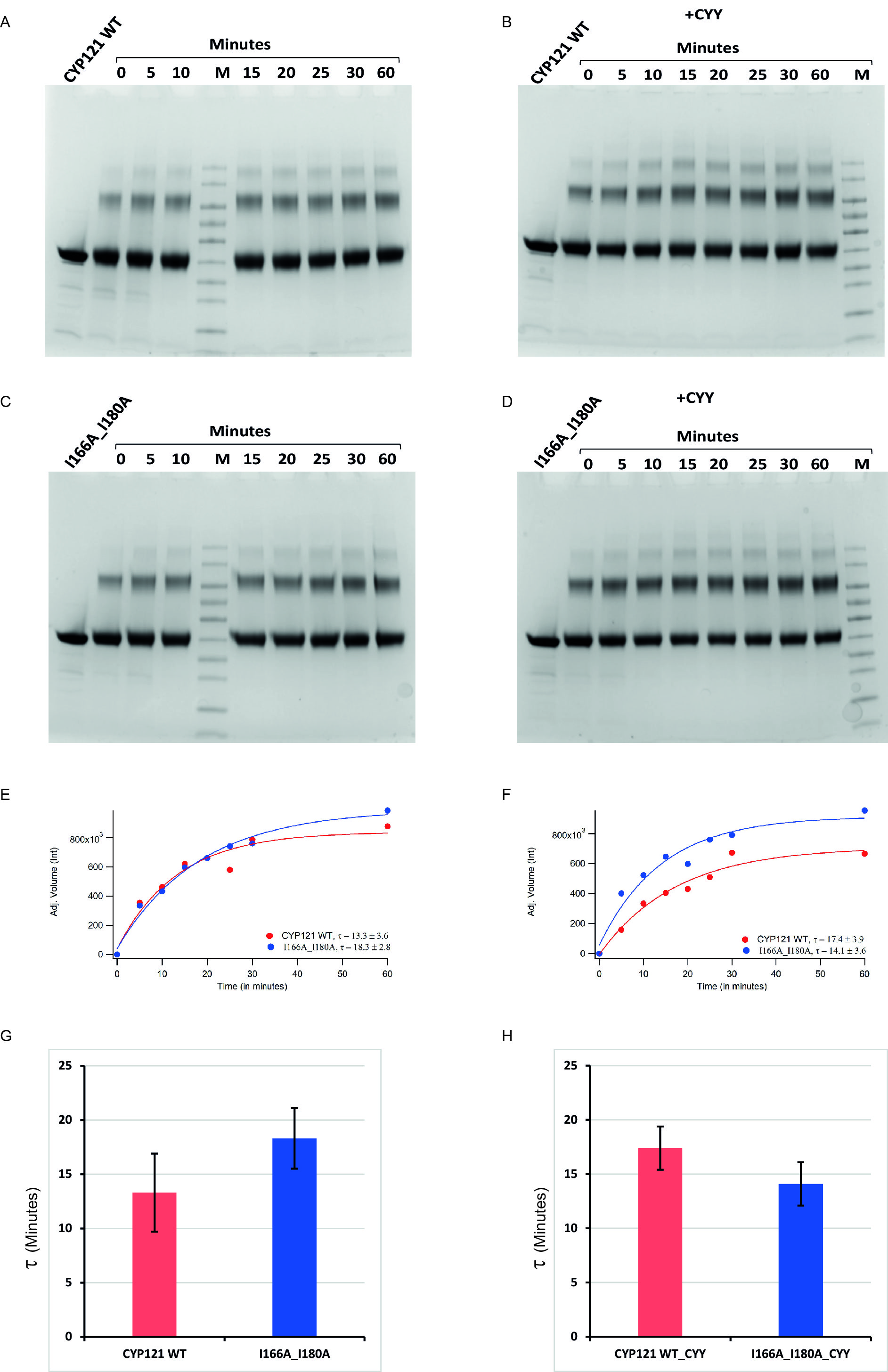
**

**Figure S3. Time-dependent crosslinking comparision between CYP121 WT (A and B) and I166A_I180A double mutant (C and D) in presence (B and D) and absence of cYY (A and C).** SDS-PAGE of crosslinked products indicates similar glutaraldehyde cross-linking efficiencies for either form. This may be due to the non-specific nature of the cross-linker, which may form covalent complexes by modification of additional surfaces besides the nonpolar dimer interface. The 50% crosslinked product saturation times were calculated by quantification of cross-linked products (Image Lab) as fraction of total protein and fitting the halfway times to a saturation curve. Gels were imaged using Bio-Rad ImageLab Version 6.1 (https://www.bio-rad.com/).


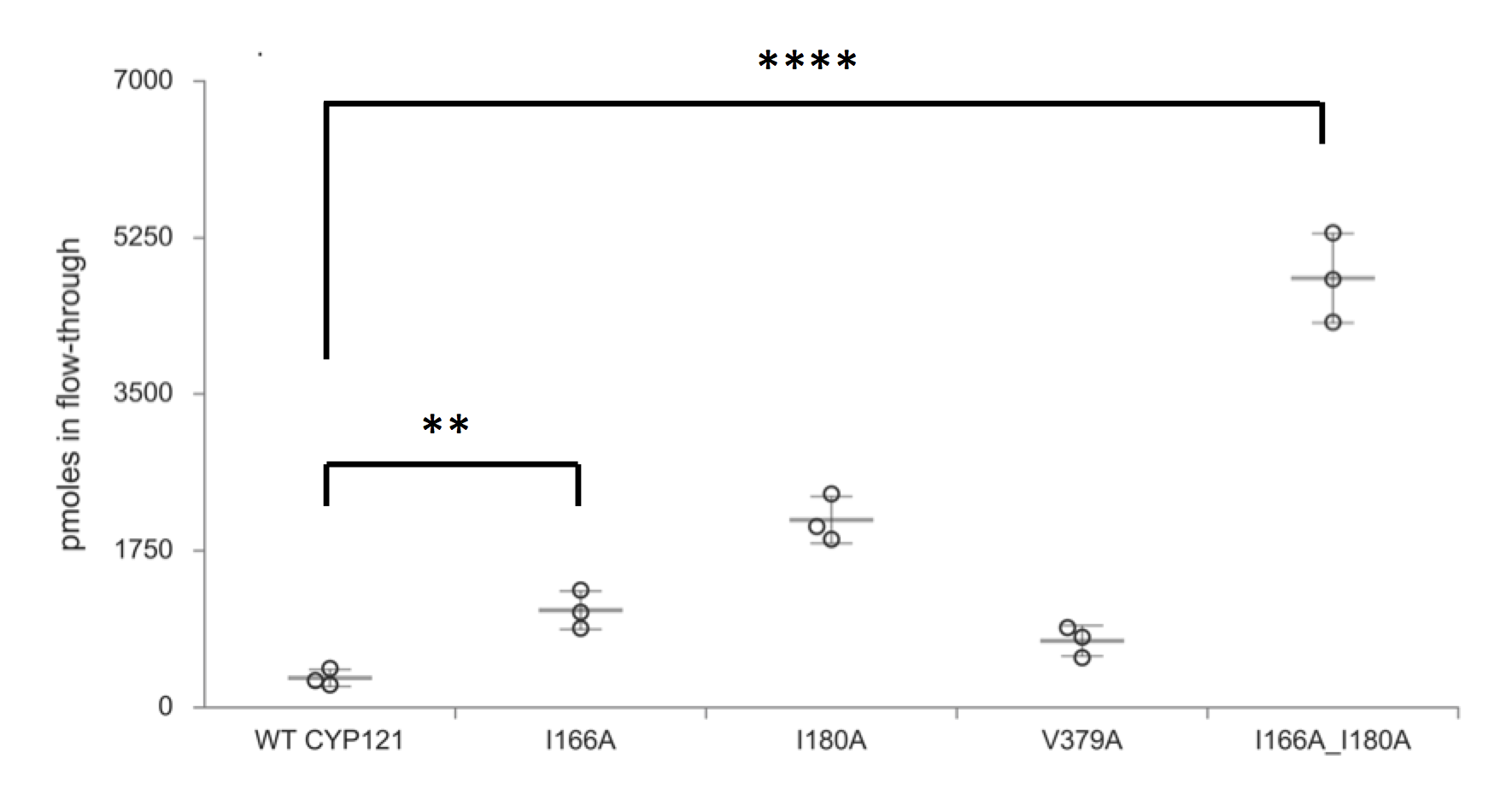


**Figure S4. CYP121 50 kDa filtration recovery assay**. Passage of CYP121A1 through a 50 kDa sieve was quantified by absorption spectroscopy. Data were analyzed for significance using one-way ANOVA. Dot plots were generated using the Interactive Dotplot server (<http://statistika.mfub.bg.ac.rs/interactive-dotplot/data/define>) and the figure generated using Adobe Illustrator Version 13.0.0 (https://www.adobe.com/).


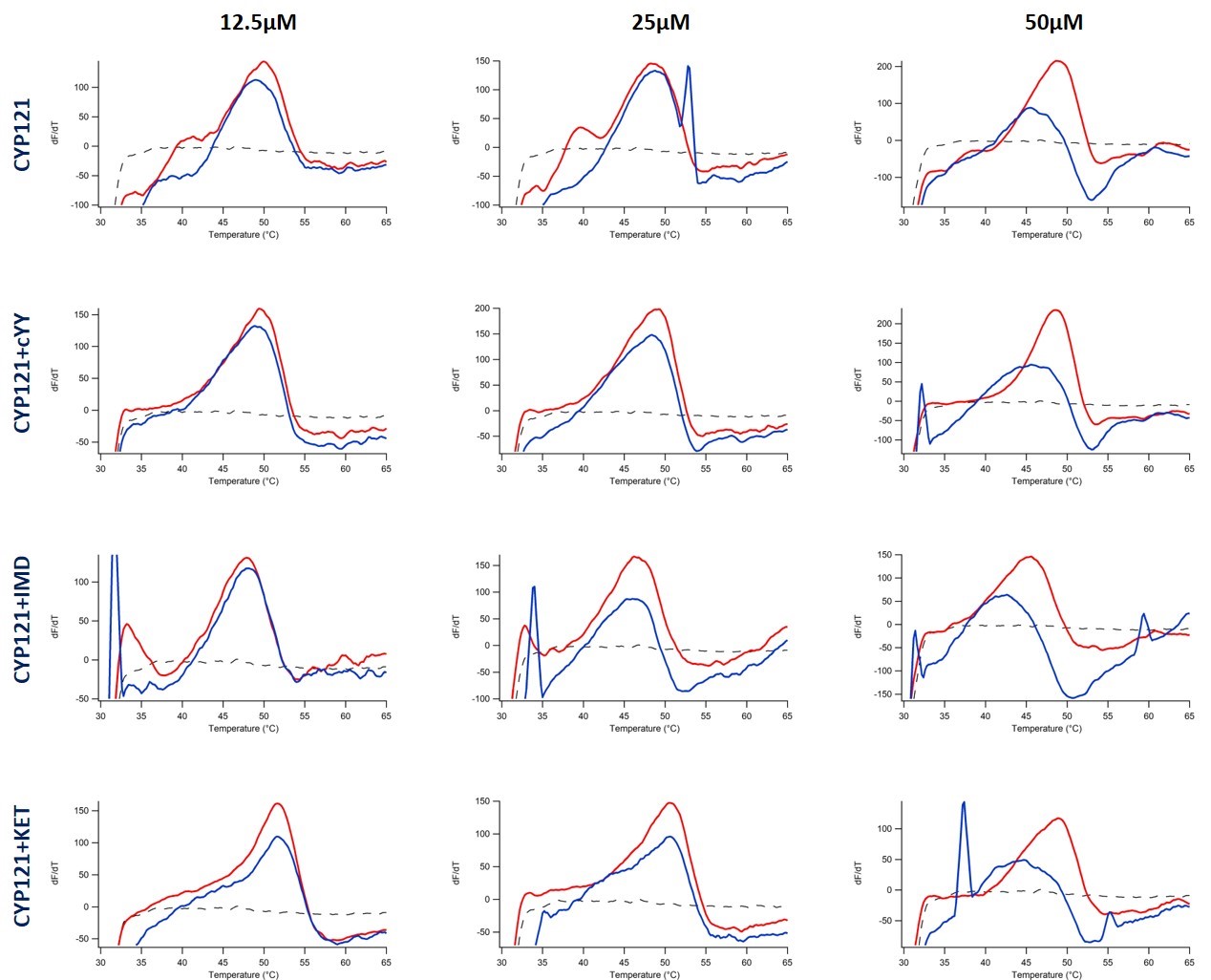


**Figure S5. Concentration-dependent Tm shift of wild type CYP121A1 and** **mutant (I166A_180A) in the presence of ligand.** Comparative thermograms showed the effect of concentration (horizontal panels) on the shape of thermogram and the effect of ligands on the thermal peak (vertical panel) where cyclo-L-Tyr-L-Tyr (cYY), imidazole (IMD), and ketoconazole (KET) were used. The red traces are for wild type CYP121A1 and blue traces represent mutant (I166A_180A). Thermal traces were generated using Igor Pro Version 6.37 (https://www.wavemetrics.com/) and graphics designed by Adobe Illustrator CS5 (https://www.adobe.com/).

**
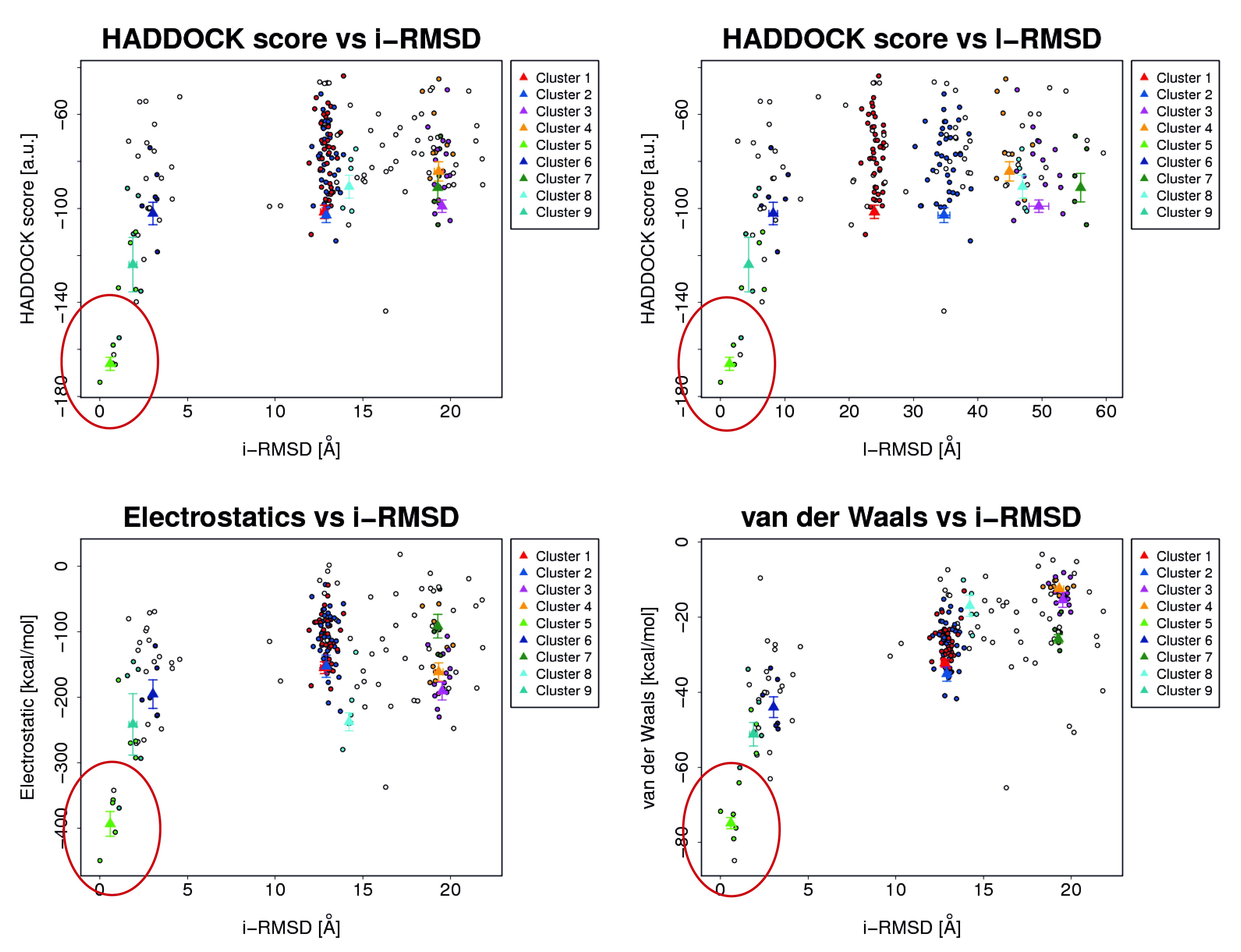
**

**Figure S6. Distribution of HADDOCK protein docking clusters of CYP121A1 dimers.** Solution clusters are plotted by HADDOCK score, (http://milou.science.uu.nl/services/HADDOCK2.2), Electrostatic, and van der Waals energy calculations versus interfacial (i) and ligand root-mean-square deviations. i-RMSD values between 0 and 1 are indicative of an energetically favorable dimer arrangement. Clusters 5, 6, and 9 all represent a distal-to-distal arrangement that reflects the FG-loops oriented on opposite sides of the complex. Of these, cluster 5 (circled) was used in all follow-on structural analyses. Docking plots were generated using Adobe Illustrator Version 13.0.0 (https://www.adobe.com/).
